# Exploration of the structural requirements of Aurora Kinase B inhibitors by a combined QSAR, modelling and molecular simulation approach

**DOI:** 10.1038/s41598-021-97368-3

**Published:** 2021-09-21

**Authors:** Sajda Ashraf, Kara E. Ranaghan, Christopher J. Woods, Adrian J. Mulholland, Zaheer Ul-Haq

**Affiliations:** 1 Dr. Panjwani Center for Molecular Medicine and Drug Research, International Center for Chemical and Biological Sciences, University of Karachi, Karachi, 75270 Pakistan; 2 Centre for Computational Chemistry, School of Chemistry, University of Bristol, Bristol, BS8 1TS UK

**Keywords:** Biophysics, Computational biology and bioinformatics

## Abstract

Aurora kinase B plays an important role in the cell cycle to orchestrate the mitotic process. The amplification and overexpression of this kinase have been implicated in several human malignancies. Therefore, Aurora kinase B is a potential drug target for anticancer therapies. Here, we combine atom-based 3D-QSAR analysis and pharmacophore model generation to identify the principal structural features of acylureidoindolin derivatives that could potentially be responsible for the inhibition of Aurora kinase B. The selected CoMFA and CoMSIA model showed significant results with cross-validation values (q^2^) of 0.68, 0.641 and linear regression values (r^2^) of 0.971, 0.933 respectively. These values support the statistical reliability of our model. A pharmacophore model was also generated, incorporating features of reported crystal complex structures of Aurora kinase B. The pharmacophore model was used to screen commercial databases to retrieve potential lead candidates. The resulting hits were analyzed at each stage for diversity based on the pharmacophore model, followed by molecular docking and filtering based on their interaction with active site residues and 3D-QSAR predictions. Subsequently, MD simulations and binding free energy calculations were performed to test the predictions and to characterize interactions at the molecular level. The results suggested that the identified compounds retained the interactions with binding residues. Binding energy decomposition identified residues Glu155, Trp156 and Ala157 of site B and Leu83 and Leu207 of site C as major contributors to binding affinity, complementary to 3D-QSAR results. To best of our knowledge, this is the first comparison of WaterSwap field and 3D-QSAR maps. Overall, this integrated strategy provides a basis for the development of new and potential AK-B inhibitors and is applicable to other protein targets.

## Introduction

The Aurora kinase family is a group of serine/threonine kinases that regulate various aspects of mitosis including centrosome duplication, chromosomal rearrangement spindle formation, activation of mitotic checkpoint and cytokinesis^1,2^. Any kind of disturbance in these processes eventually leads to cell death or aneuploidy. Human aurora kinases are functionally and structurally categorized into three subtypes, aurora A, B and C. All three isoforms contain a conserved catalytic domain, a variable N-terminal domain, and a short C-terminal extension. Despite significant similarity among these kinases, the location and functions are different from one another^3–7^.

Aurora A is found on chromosome 20q13.2 (a region amplified in various cancers) and expressed in initial mitosis at G2/M phase. Ajuba, BRCA-1, CDC25B, Eg5, TPX2 and p53 are reported as substrates for Aurora A kinase It is necessary for multiple key events in cell division including maturation of centrosome, segregation and assembly of spindles^8,9^. It acts as an oncogene and its over expression is found in several tumours such as colon, breast, pancreatic, ovarian and bladder cancers^10–12^. Aurora B is located on 17p13 chromosome and acts as a chromosomal passenger protein^13^ along with its two substrates, INCENP and Survivin^14–16^. Each of these proteins is required for AK-B to correct chromosome location in mitosis process. Similar to AK-A, it regulates different processes in mitosis. AK-B activity is based on the phosphorylation of its residue Thr232 and Ser10 of its substrate Histone H3^15,16^. necessary for accurate completion of mitosis Inhibition of AK-B activity can lower the H3 phosphorylation, as a result of which improper condensation of chromosome and in-complete cytokinesis occurs, ultimately leading to cell apoptosis. Its over-expression is found in multiple human cancers such as mesothelioma, malignant endometrium, glioblastoma, non-small cell lung carcinoma, hepatocellular carcinoma, testicular germ cell tumours, oral cancer, thyroid, ovarian, colorectal and prostate cancer^17–20^.

Aurora kinase C particularly is found in the testes and plays an important role in spermatogenesis and regulates the movement of flagella and cilia^21^.

Structurally, Aurora kinases are comprised of three domains: the N-terminal domain, followed by a conserved kinase domain, and a C–terminal domain. The N-terminal domain exhibits sequence dissimilarity thus providing selectivity for protein–protein interactions. The kinase domain constitutes a β-stranded lobe and an α-helical lobe on the N-terminal and C-terminal respectively connected by a hinge region. The autophosphorylation of Thr 288 (AurA), Thr232 (AurB) and Thr195 (AurC) in the catalytic T-loop region of the kinase domain’s C-terminal lobe results in conformational changes thereby activating the kinase domain^22,23^. Aurora kinases are considered as potential drug targets for tumour remedy due to their strong association with tumorigenesis^24^.

A number of small molecules inhibitors against AK-B have been reported to date. A classification for such compounds has been reported by Yan and colleagues who utilized a machine learning algorithm (Self-Organizing Map and Support Vector Machine) to classify Aurora kinase inhibitors into three classes, namely dual Aurora-A and Aurora-B inhibitors, Aurora-A selective inhibitors and selective inhibitors of Aurora-B^25^. Aurora kinases are effectively inhibited in numerous preclinical cell line and animal models. Several small molecule inhibitors have been developed and are indifferent stages of clinical trials. Uptil now only ten clinical trials have been reported, using inhibitors targeting specifically AK-B and most of them are still in phase I stage^26^ such as AK-B selective inhibitors Barasertib (AZD1152)^27–31^, Chiauranib^32,33^, BI847325^34^, PF-03814735^35,36^, GSK1070916^37–39^, TAK-901^40^ and hesperidin^41^ and the pan Aurora A/B inhibitors VX-680^19^ and ZM447439^17–19,29^. Most of these inhibitors are ATP-competitive with a planar heterocyclic ring system that can reside in the adenine-binding region and mimic adenine–kinase interactions. These inhibitors are derivatives of indole, bis-indole, pyrrolopyrazole, pyrimidine, thiazolo-quinazoline, quinazolin, pyrazoloquinazoline, fused tricyclic structures, and some other structures^42^. In addition, the prospect of designing potent allosteric inhibitors for AK-B appears very promising from the clinical perspective, because it may generate selective inhibitors^43^. Recently, Colombo and colleagues proposed a new possibility in pursuit of designing selective chemical tools by perturbing the intermolecular interaction between chaperone and kinases thus, targeting protein folding and rendering it inactive^44^. Unfortunately, despite intensive efforts, no small molecule inhibitor of AK-B has been approved by the FDA, thus demanding the development of new inhibitors.

In the current study, a multiplex computational approach including molecular docking, 3D-QSAR, pharmacophore-based screening, MD simulation and binding free energy calculation was performed to investigate the binding mechanism of AK-B inhibitors. The 3D-QSAR model was built using CoMFA and CoMSIA approaches to explore the structural requirements of inhibitors impacting their inhibitory activities. Furthermore, to investigate the conformation and contribution of key residues to the potency of compounds, docking, MD simulations and binding free energy calculations were performed. In this study our main focus is to demonstrate a combined computational approach: the combination of QSAR with simulation, specifically in combination with the WaterSwap^45^ method. This is first such combined application according to best of our knowledge. By understanding the binding mode of aurora kinase B inhibitors, we can get deeper insights into the structural requirements that may produce more selective and potential AK-B inhibitors.

## Materials and methods

### Data set

A structurally diverse dataset of 57 compounds was collected from literature to perform 3D-QSAR studies probing the effect of inhibition against AK-B^46,47^. On the basis of structural diversity and activity, 45 out of 57 compounds were randomly selected as the training set however, the remaining 12 compounds were used as the test set. The training set was used to build the 3D-QSAR model while the test set compounds were used for the verification of the developed models. The test compounds evenly covered the range of biological activity and structural diversity of the dataset. Observed biological activities (IC_50_) were changed to negative logarithm (pIC_50_) before 3D-QSAR model generation. The chemical structures and the experimental activities of compounds are presented in Table S1 (supplementary material).

### Structure preparation

The crystal structures of Aurora kinase B crystallized with inhibitors were taken from the protein data bank (Table 1). The structure preparation was performed in MOE^48^ and the missing loops, steric clashes and missing atom names were corrected. The hydrogen atoms were added, water molecules were removed, and the energy minimization was performed using Amber99 force field. The protonation state of every titratable residue within the complexes was assigned at physiological conditions using the Protonate-3D module of MOE^48^ and their conformations were produced with default parameters by using the Conformation Search option. Finally, the protein complex was minimized (giving a RMSD of 0.3 Å by selecting heavy atoms) using the force field AMBER99^49,50^.

**Table 1:** X-ray crystal complex structures of AK-B^51–55^.

Structure no.	PDB ID	Resolution
1	4AF3	2.75 Å
2	2BFY	1.8 Å
3	2VGO	1.7 Å
4	2VGP	1.7 Å
5	4B8M	1.85 Å
6	4C2V	1.49 Å
7	4C2W	1.7 Å
8	3ZTX	1.95 Å

### Pharmacophore modeling

The MOE option in LigandScout^56^ was used to build the pharmacophore models on the basis of available crystal structures of AK-B complex (Table 1). Hydrophobic (HY), hydrogen-bond donor (HBD), hydrogen-bond acceptor (HBA) and exclusion volume features were considered for the generation of pharmacophore model. Numerous pharmacophore models were generated with significant statistical parameters. The best model was designated on the basis of a good correlation coefficient (r) and rmsd values. Moreover, the developed model was further validated by actives, decoys and random sets of molecules.

### Molecular docking

The MOE software package (Molecular Operating Environment, 2015.01) was used to perform docking. The crystal structure of human AK-B complex (PDB ID: 4AF3) was retrieved from the Protein Data Bank^51^ and used for docking studies as it is the only available crystal structure of human Aurora kinase B. The generated conformations were minimized using the MMFF94 × force field (Merck Molecular Force Field 94 × gradient: 0.05 kcal/mol). The Triangle Matcher placement method was used for molecular docking with London dG and Generalized-Born Volume Integral/Weighted Surface area (GBVI/WSA) scoring function^48^. All compounds presented in Table S1 were docked successively in the binding cavity of AK-B using default parameters. The lowest score docked pose of every compound was considered for protein–ligand interactions and QSAR modeling.

### CoMFA and CoMSIA analysis

The CoMFA^57^ potential fields (steric and electrostatic) were calculated at every point of regular grid spacing lattice of 2.0 Å. A probe of sp^3^ carbon atom was used to calculate the electrostatic and steric field energies with contributions trimmed to 30 kcal/mol and the remaining default parameters were used. For CoMSIA^58^ studies, five different descriptor fields such as electrostatic (E), steric (S), hydrophobic (H), hydrogen bond donor (HBD) and acceptor (HBA) were calculated. Descriptors used for CoMSIA were obtained by the same lattice box as used for CoMFA method. Both CoMFA and CoMSIA methods use the same function depicted by Gaussian function and raises the hydrogen bond donor, acceptor and hydrophobic fields. CoMSIA results illustrate a comparatively small effect from molecule corresponding rules and can more instinctively clarify a compound's SAR relationship. The intrinsic defects of CoMFA method can be overcome by CoMSIA method but this does not essentially obtain better results^59^. Thus, in the current study, CoMFA and CoMSIA methods can validate and complement each other to achieve consistent predicted models.

### Partial least square analyses (PLS)

To associate the CoMFA or CoMSIA fields of 3D-QSAR utilizing AK-B inhibitors and their biological activities, partial least square (PLS) analysis (regression analysis) was performed. The optimal number of components that produces most promising extrapolative models was recognized by using cross-validation leave-one-out (LOO) method. The column filtering value was set at 2.0 kcal/mol to make better signal to noise ratio, by removing some lattice points, with less difference in energy than threshold value. The PLS analysis was performed using no column filtering with non-cross-validation method. The mean values for cross-validation correlation coefficient q^2^ and the resultant standard error of prediction (SEP) values were achieved by reiterating every random run of cross-validation.

### Validation of CoMFA and CoMSIA models

The robustness and internal prediction power of the generated models was assessed by cross-validation leave one out (LOO) method. The q^2^ and r^2^ values showed the internal prediction power and sturdiness of the model respectively. The average values of r^2^ (r_boot_^2^) and SEE (SEE _boot_)) were calculated, a bootstrapping analysis of 60 runs was also performed to measure the biasness of the calculation. It has been reported that only q^2^ value is not enough to assess the predictive quality of 3D-QSAR model thus external validation must be applied. For this purpose, a total of eleven test set compounds that were not utilized previously to build the QSAR model were used for the external validation. The external test set validation is considered as the most reliable validation method to evaluate the extrapolative capability of the developed model. The predictive ability of CoMFA and CoMSIA models was assessed externally by forecasting the biological activity of independent set of test compounds. The model prediction power was expressed by r^2^ (rpred^2^ > 0. 6), variance in prediction (Qext^2^ > 0. 5), standard error of prediction (SEP) and external standard deviation error of prediction (SDEPext).

The given equation was used to calculate $R_{pred}^{2}$^.^

1 $$R_{pred}^{2} = \frac{{1 - {\text{PRES}}}}{{{\text{SD}}}}$$

where PRES indicates the sum of squared deviations between biological and predicted activity values while SD indicate the sum of squared deviations between average biological activity of compounds in the training set and experimental activity of the test set compounds.

The cross-validated coefficient (q^2^) was calculated by the following given formula:

2 $$Q^{2} = 1 - \frac{{\sum \left( {y_{i } - \tilde{y}_{i} } \right)}}{{{\Sigma }\left( {y_{i } - \overline{y}} \right)^{2} }}$$

where *y*_*i*_ and $\tilde{y}_{i}$ are the experimental and the predicted values, respectively; and $\overline{y}$ is the averaged value for the variables of the training set.

### Molecular dynamics simulations

To test the stability of the docked complexes, and for insight into the binding interactions in the inhibitor complexes, molecular dynamic simulations were performed with the AMBER 12 simulation software package^49^ using the ff14SB^60^ and ff99SB force field^61^. The Acyl-54 (most active), Acyl-24 (inactive), ZINC11253730, ZINC42019540, ZINC65618522 and ZINC07046484 compounds bound in target structures were obtained from molecular docking, as described above. The atomic charges of the compounds were calculated based on the electrostatic potential from single point HF/6-31G* calculations using Gaussian 03 and fitted using RESP in the Antechamber module^62^. The GAFF force field^63^ was used to parametrize the compounds. LEaP module was used to assign Hydrogen atoms by setting default protonation states of ionizable residues at a neutral pH. The systems were solvated using the TIP3P water model and a minimum of 10 Å solute-wall distance was used to cover every complex. Each complex was neutralized with chloride counter ions. The solvated systems were energy minimized by following two stages of steepest descent algorithm and conjugate gradient algorithm. The molecular dynamic simulations were performed using heating, density equilibration and production run in Berendsen isothermal isobaric ensemble MD. The systems were gradually heated up to 300 K in 500 ps at constant volume and constant pressure (1 atm) with a Langevin thermostat that was used to maintain a temperature of 300 K. While an isotropic pressure scaling algorithm was used to maintain the pressure of 1 bar, using a pressure relaxation time of 1 ps. Then every system underwent production run of 200 ns. The particle mesh Ewald method was used for calculating long-range electrostatic interactions, with a 10 Å cutoff. The SHAKE algorithm was used to constrain bonds involving hydrogen atoms to their equilibrium length. After equilibration, coordinates were saved every 50 ps. These collected trajectories were used for structural and energetic analysis of each system.

### Binding free energy calculations

Binding free energy of all systems was calculated by two different methods: WaterSwap^45,64^ and MM(PB/GB)SA^65^. WaterSwap is an absolute binding free energy method that avoids the cavitation and some other issues of double-decoupling methods^45^. WaterSwap calculates the free energy for exchanging a bound ligand with a flexible water cluster of similar shape and size. The free energy difference is calculated by Monte Carlo replica exchange simulations along the Water Swap Reaction Coordinate. It uses an explicit treatment of water, so includes the detail of protein–water, protein–water–ligand and ligand–water interactions that are missing in continuum solvent methods^64^. WaterSwap uses a reaction coordinate which swaps the bound ligand with an equivalent volume and shape of water, moving the ligand from the protein into bulk solvent, with simulations at points along this WaterSwap Reaction Coordinate, using a dual topology algorithm. It uses an identity constraint^66^ to define the water cluster: this identity constraint places identity points in space instead of labelling water molecules to define water molecules in the binding site. The absolute binding free energy is calculated by replica exchange thermodynamic integration method^67^. The end points are a pair of simulation boxes (coupled to the same thermostat), one being a water box (box of water molecules) and the other is protein box contain protein–ligand complex solvated in periodic boundary box of explicit water molecules.

With the identified cluster, the total energy of the system is assessed using the following equation,

3 $$\begin{aligned} {\text{E }}\left( \lambda \right) \, & = {\text{ E}}_{{{\text{proteinbox}}}} + {\text{ E}}_{{{\text{waterbox}}}} + {\text{E}}_{{{\text{ligand}}}} + {\text{E}}_{{{\text{cluster}}}} + \, \left( {{1 } - \, \lambda } \right) \, \left( {{\text{E}}_{{\text{ligand:proteinbox}}} + {\text{ E}}_{{{\text{cluster}}:{\text{waterbox}}}} } \right) \\ & \quad + \, \left( \lambda \right) \left( {{\text{E}}_{{{\text{cluster}}:{\text{proteinbox}}}} + {\text{ E}}_{{{\text{ligand}}:{\text{waterbox}}}} } \right) \\ \end{aligned}$$

where *E*_proteinbox_ is the energy of all the molecules in the protein box except the ligand, *E*_waterbox_ is the energy of all the molecules except the water cluster identified in the water box, *E*_ligand_ represents the intramolecular energy of the ligand, *E*_cluster_ is the intermolecular energy between all the water molecules present in the water cluster, *E*_ligand:protein box_ is the energy of interaction between the ligand and all atoms of the protein box, *E*_cluter:water box_ is the resultant energy of interactions between all water molecules and water clusters of the water box, λ is the WaterSwap Reaction Coordinates, used to scale *E*_cluster:water box_ by 1 − λ.

The decoupling of the ligand from the protein box is associated with decoupling of water cluster from water box. Simultaneously, cluster of water coupled to the protein box and ligand is coupled to the water box. This energy calculation between water molecules and the ligand in the protein box is represented by *E*_ligand:water box,_ and the molecules and water cluster in the box is presented as *E*_cluster:protein box_ and scaling by λ. The λ is a single coordinate reaction that is changed from λ = 0 (ligand bound to the protein in protein box) to λ = 1 (unbound ligand and is in bulk water). Moreover, it corresponds to a transferred of water cluster to the protein box for filling the resulting cavity.

The absolute binding free energy is calculated by thermodynamic integration (TI) using the gradient of energy calculations with respect to λ,

4 $${\text{dE}}/{\text{D }}\lambda \, = \, \left( {{\text{E}}_{{{\text{cluster}}:{\text{proteinbox}}}} + {\text{E}}_{{{\text{ligand}}:{\text{waterbox}}}} } \right) \, {-} \, \left( {{\text{E}}_{{{\text{ligand}}:{\text{waterbox}}}} + {\text{E}}_{{{\text{cluster}}:{\text{waterbox}}}} } \right)$$

The ensemble average is calculated at different values of λ to obtain free energy gradient across λ,

5 $${\text{dG}}/{\text{d}}\lambda \, = {\text{ dE}}/{\text{d}}\lambda$$

To get average free energy gradients, Monte Carlo (MC) sampling for each λ must be achieved. The binding free energy is finally obtained by integrating the gradient across λ,

6 $${\text{G}}_{{{\text{bind}}}} = \int_{0}^{1} {\left( {{\text{dG}}/{\text{d}}\lambda } \right)} {\text{ d}}\lambda$$

WaterSwap calculations of absolute binding free energy were performed with the Sire package using the WSRC module^45^, these calculations used the same forcefield and solvent model as in the molecular dynamics simulations^67^. For each system, five WaterSwap calculations were performed of absolute binding free energies. The starting structures for WaterSwap calculation taken from clustering of the 100 ns trajectories. Errors were calculated from standard errors on these averages. The WaterSwap binding free energies were calculated by replica-exchange thermodynamic integration over 16 λ windows (0.005, 0.071, 0.137, 0.203, 0.269, 0.335, 0.401, 0.467, 0.533, 0.599, 0.665, 0.731, 0.797, 0.863, 0.929, 0.995) across the WaterSwap Reaction Coordinate. For each window, 50 million Monte Carlo steps were performed, and absolute free energies calculated by Free Energy Perturbation (FEP), Thermodynamic Integration (TI), and Bennetts algorithm, from the free energy gradient calculated over the last 30 million steps. The “Set A” soft-core parameters are used in the simulations with 15 Å Lennard–Jones and coulomb non-bonded cutoff, the shifted force field used to account long range electrostatics and reflection sphere used to restrict sampling within 15 Å radius of compound. VMD 1.9.1^68^ used to visualize the trajectories. Analysis and visualization were carried out with alignment of protein backbone using RMSD tool in VMD.

The binding free energy of the system was also calculated separately by a completely different approach, namely the MM(PB/GB)SA method implemented in AMBER 16^69^. This is an implicit solvent approach, which can give useful results in some cases. 1000 frames were extracted from the simulation trajectories and MMPBSA.py module of AMBER was used for calculation analysis. In the MM(PB/GB)SA approach, the binding free energy is calcualted as the difference between the free energy of complex, and that of the ligand and receptor.

7 $$\Delta {\text{G}}_{{{\text{binding}}}} = {\text{G}}_{{{\text{complex}}}} {-}{\text{G}}_{{{\text{protein}}}} {-}{\text{ G}}_{{{\text{ligand}}}}$$

Furthermore, the binding free energy was decomposed into residue contributions to identify amino acids that contribute to binding affinity^70,71^. The binding interaction of each inhibitor–residue pair includes four terms:

8 $$\Delta {\text{G}}_{{{\text{inhibitor}} - {\text{residue}}}} = \, \Delta {\text{G}}_{{{\text{vdw}}}} + \, \Delta {\text{G}}_{{{\text{ele}}}} + \, \Delta {\text{G}}_{{{\text{pol}}}} + \, \Delta {\text{G}}_{{{\text{nonpol}}}}$$

where the van der Waals contribution (ΔG_vdw_) and the electrostatic contribution (ΔG_ele_) are calculated using the Pmemd.cuda program^72^ in Amber 16. The nonpolar solvation contribution (ΔG_nonpol_) is the contribution of nonpolar to the solvation free energy and calculated from the solvent accessible surface area (SASA) model by the LCPO method: (ΔG_SA_ = 0.0072 × ΔSASA). The polar solvation contribution (ΔG_pol_) was computed using the generalized Born module in Amber16.

## Results and discussion

### Molecular docking

**Figure 1:**
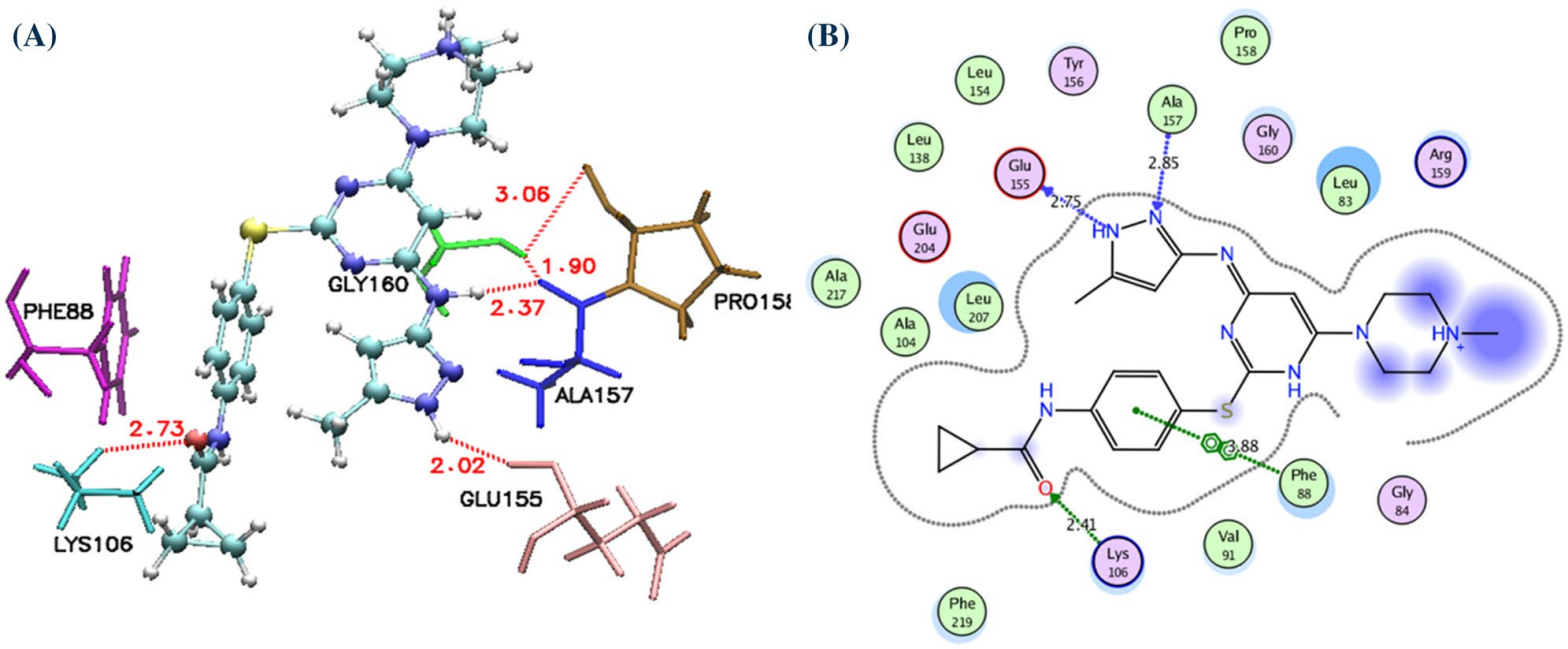
Docking of compounds into the binding site of Aurora kinase B. (**A**) Compound VX-680, (**B**) Acyl-54. Compounds and active site residues for Ligands and the important residues for binding interaction are represented by stick and line models 1 (**A**), while in 1 (**B**) non-polar and polar residues are presented in green and pink circles; hydrogen bond interaction is indicated by green dotted arrows; the shape of the binding site indicated by the proximity contour (dotted lines) surrounding the ligand.

Before starting the docking, all available crystal structures of AK-B protein were assessed to evaluate the degree of similarity in their binding sites. The results indicate that most of the binding site residues are conserved within the binding site (Figure S1). After evaluating the degree of similarity, all compounds were docked to the binding site of 4AF3 protein. The docking produced 30 conformations for each compound. The clusters were examined, and the final docked conformation was selected on the basis of scores. The binding pocket of AK-B is composed of residues Leu83, Phe88, Val91, Ala157 and Leu207. The docked conformation of VX-680, which is a cognate ligand of aurora kinase B, is shown in the Fig. 1. It was observed that compound acyl-54 was well located within the binding pocket of AK-B and showed similar type of interaction with the active site residues of AK-B as presented by cognate ligand. The amino group of Ala157 forms two hydrogen bonds with pyrazolidine and linker nitrogen of VX-680 at a distance of 1.92 and 2.39 Å. Additionally Leu83, Val91 and Phe88 are involved in hydrophobic interaction while Phe88 forms pi–pi interaction with the benzene ring of VX-680. In case of acyl-54, a hydrogen bonding interaction is found between amino group of Ala157 and carbonyl group of acyl-54 at a distance of 1.85 Å. Another hydrogen bond is found between Glu155 and NH group of Acyl-54 at a distance of 3.05 Å. These hydrophilic interactions have been already reported in the literature^73^. As well as docking with the 4AF3^51^ structure, the selected compounds were also docked to the active site of other AK-B crystal structures (Table 1). Further, these docked conformations were used in receptor-guided 3D-QSAR studies.

### Molecular alignment

**Figure 2:**
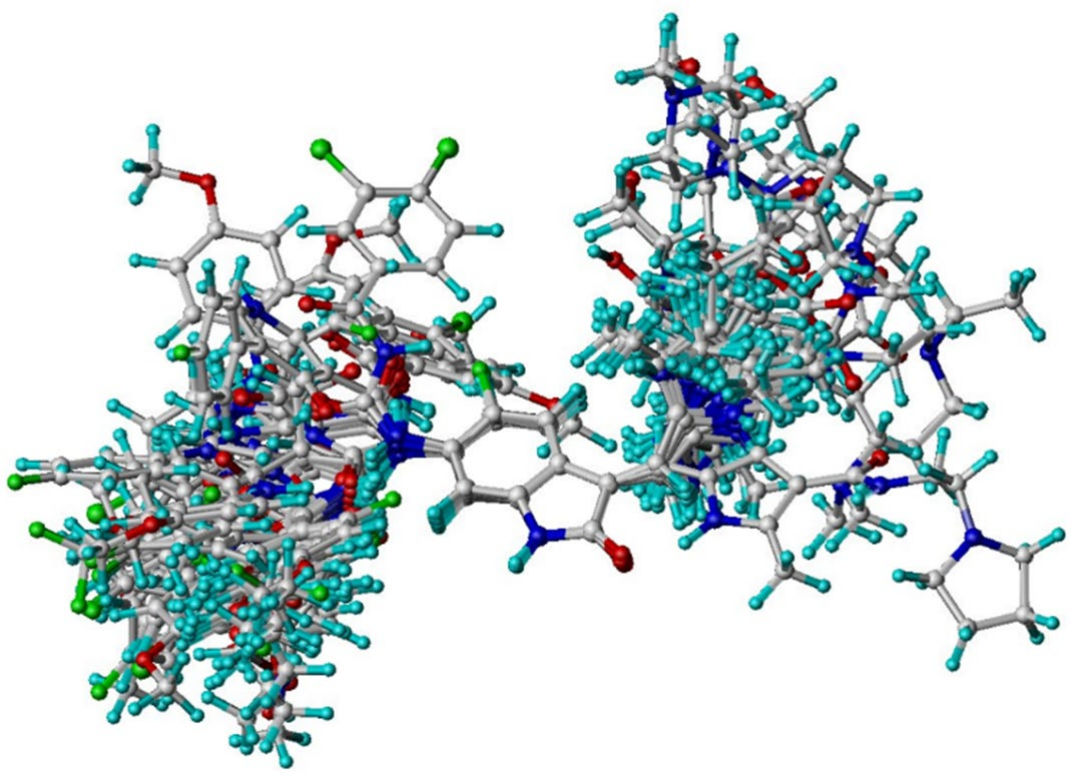
The alignment of all acylureidoindolin derivatives.

In CoMFA and CoMSIA studies, 3D structures of the molecules are required to be aligned based on a suitable conformational template and its substructure, which taken as a “bioactive” conformation. Molecular alignment based on the common scaffold has been extensively used^74,75^; conversely it is thought to be more reasonable if the model is developed and evaluated on the active conformations. Moreover, the alignment based on docked conformations will help in the contour map analysis of the models in a structure-based manner. In the present study, molecular alignment was achieved via molecular docking (Fig. 2). As a result, all the compounds were aligned in the active site for building 3D-QSAR models.

### CoMFA and CoMSIA statistics

**Figure 3:**
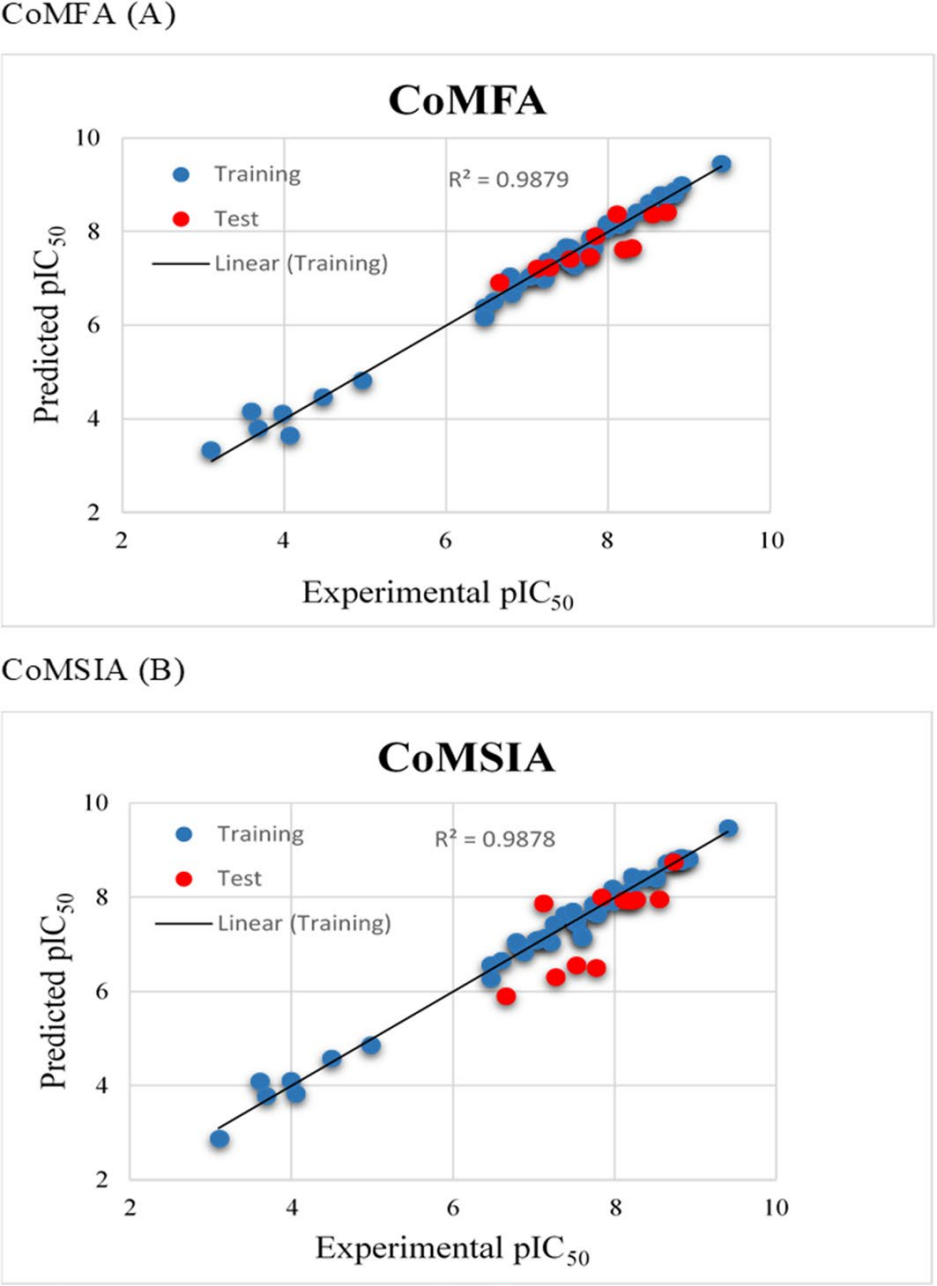
Scatter plots of experimental versus predicted pIC_50_ for the optimal (**A**) CoMFA and (**B**) CoMSIA model of training and test set.

The CoMFA and CoMSIA models were built using Sybyl7.3 to assess the changes in three-dimension structural features of chemical substitution affects the anticancer activity of acylureidoindolin derivatives. The statistical values of these models are summarized in Table 2. These statistics obtained after omitting three outliers Acyl-43, Indoline-24B and Indoline-25H. The cross-validation coefficient value of 0.68 was achieved for CoMFA model with optimum number of component 6 having standard error of prediction 0.021 while non-cross validation coefficient analysis was done with 6 number of optimal component in conventional r^2^ of 0.971, a standard error of estimation (SEE) value of 0.203 and F value of 253.86. The resultant field contributions were 63.6% and 36.4% for steric and electrostatic field respectively, displaying a higher effect of the steric field. As compared to CoMFA, CoMSIA model based on five different fields (steric, electrostatic, hydrophobic, hydrogen bond donor and acceptor fields) gave a q^2^ (cross validation coefficient) value of 0.641 and r^2^ value of 0.933 with 6 optimum number of components, standard error of estimation (SEE) of 0.2110 and F value of 122.544. The field contributions of electrostatic, steric, hydrophobic, donor and acceptor fields were 0.252, 0.160, 0.189, 0.101 and 0.298, respectively. These values indicate that hydrophobic field made greater contribution in ligand–protein binding and inhibitory activity of AK-B. Our developed models predicted the correlation coefficients r^2^_pred_ values 0.733 and 0.947, respectively. Relatively, the CoMSIA model presented better competency in predicting the biological activity of external test set compounds (Fig. 3). The statistical results indicate that these developed models are robust enough to design novel and potent inhibitors. Observed and predicted pIC50 values of CoMFA and CoMSIA model was shown in Table 3.

**Table 2:** Statistical parameters of 3D-QSAR model for internal validation.

PLS statistical parameters	CoMFA	CoMSIA
q2cv (LOO)	0.624	0.577
ONC	6	6
r2ncv	0.924	0.95
F-value	311.64	265.37
**Fraction of field contribution**		
Steric	0.406	0.118
Electrostatic	0.594	0.335
Hydrophobic	0.219	
Hydrogen bond donor	0.168	
Hydrogen bond acceptor	0.16

**Table 3:** Compounds with their reported pIC_50_ and predicted pIC_50_ by CoMFA and CoMSIA Model along with their residuals.

Compound no.	pIC_50_	CoMFA predicted	Residual	CoMSIA predicted	Residual
**Training set of the best model**					
Indoline11A	7.6	7.26	0.34	7.196	0.4
Indoline11B	7.53	7.651	− 0.12	7.501	0.03
Indoline12	8.23	8.211	0.02	8.436	− 0.21
Indoline13	7.03	7.027	0	7.07	− 0.04
Indoline14	6.6	6.506	0.09	6.653	− 0.05
Indoline14B	6.46	6.381	0.08	6.561	− 0.1
Indoline24A	3.1	3.331	− 0.23	2.876	0.22
Indoline24C	4.06	3.644	0.42	3.826	0.23
Indoline24D	3.68	3.792	− 0.11	3.778	− 0.1
Indoline24G	3.6	4.164	− 0.56	4.091	− 0.49
Indoline24H	3.99	4.115	− 0.12	4.102	− 0.11
Indoline24I	4.49	4.463	0.03	4.574	− 0.08
Indoline25A	4.97	4.816	0.15	4.858	0.11
Indoline31A	6.89	6.874	0.02	6.824	0.07
Indoline31B	7.99	8.163	− 0.17	8.185	− 0.19
Indoline31C	7.48	7.668	− 0.19	7.695	− 0.22
Indoline31D	7.2	6.987	0.21	7.037	0.16
Indoline31F	6.46	6.171	0.29	6.265	0.2
Indoline31G	6.79	7.045	− 0.25	7.05	− 0.26
Indoline31H	7.59	7.533	0.06	7.137	0.45
Indoline32	8.35	8.407	− 0.06	8.393	− 0.04
Indoline33	7.15	7.184	− 0.03	7.141	0.01
Indoline34	7.04	7.045	− 0.01	7.095	− 0.06
Acyl-32C	8.19	8.215	− 0.03	8.139	0.05
Acyl-32F	7.39	7.487	− 0.1	7.623	− 0.23
Acyl-32G	7.82	7.647	0.17	7.826	− 0.01
Acyl-32H	7.74	7.513	0.23	7.673	0.07
Acyl-32I	7.53	7.336	0.19	7.463	0.07
Acyl-32J	6.8	6.665	0.13	6.974	− 0.17
Acyl-35	7.26	7.365	− 0.11	7.424	− 0.16
Acyl-38	8.74	8.769	− 0.03	8.781	− 0.04
Acyl-39	8.92	8.991	− 0.07	8.808	0.11
Acyl-40	8.82	8.798	0.02	8.757	0.06
Acyl-41	8.52	8.614	− 0.09	8.427	0.09
Acyl-42	8.64	8.78	− 0.14	8.724	− 0.08
Acyl-44	8.52	8.528	− 0.01	8.385	0.14
Acyl-45	8.82	8.866	− 0.05	8.836	− 0.02
Acyl-48	7.51	7.454	0.06	7.447	0.06
Acyl-49	7.74	7.572	0.17	7.833	− 0.09
Acyl-51	7.79	7.86	− 0.07	7.638	0.15
Acyl-52	7.98	8.052	− 0.07	7.895	0.09
Acyl-53	8.12	8.129	− 0.01	8.065	0.05
Acyl-54	9.4	9.45	− 0.05	9.467	− 0.07
**Test set compounds**					
Indoline31E	7.27	7.228	0.04	6.304	0.97
Indoline31I	7.53	7.413	0.12	6.554	0.98
Indoline35	8.54	8.358	0.18	7.953	0.59
Indoline36	7.13	7.208	− 0.08	7.864	− 0.73
Acyl-32A	7.83	7.902	− 0.07	7.997	− 0.17
Acyl-32B	8.28	7.65	0.63	7.944	0.34
Acyl-32D	8.11	8.366	− 0.26	7.934	0.18
Acyl-32E	8.19	7.616	0.57	7.915	0.27
Acyl-34	8.72	8.412	0.31	8.741	− 0.02
Acyl-47	6.66	6.907	− 0.25	5.896	0.76
Acyl-50	7.77	7.456	0.31	6.5	1.27

### CoMFA and CoMSIA model analysis based on predicted pIC_50_ of AK-B inhibitors

The CoMFA and CoMSIA model results are summarized in Table 2. The generated 3D-QSAR models were found to be reliable and satisfactory if q^2^ value is greater than 0.50 and r^2^ value is greater than 0.90, These statistical keys, representing that both models have a robust extrapolative power by demonstrating a good correlation between the predicted and experimental log IC_50_ values. The stability and robustness of the model is further confirmed by bootstrapping technique for 60 runs. The average r^2^ (r^2^_boot_) and SEE (SEE _boot_) of these analyses of CoMFA and CoMSIA model are 0.9649, 0.013 and 0.941, 0.021 respectively. The greater value of r^2^_boot_ and lowest value of SEE describe the robustness of generated CoMFA and CoMSIA model. Further robustness of these model was tested by shuffling the log IC_50_ values. The resulting q^2^ and r^2^ values were in the range of − 0.059 to 0.085 and − 0.006 to 0.030 respectively. The predicted pIC_50_ values of acyl and indoline derivatives are listed in Table 3. The correlations between calculated and predicted pIC_50_ values for the training and the test sets are depicted in Fig. 3. External validation (using a test set) was also carried out to evaluate the stabilities and the predictive capability of the generated QSAR model. These statistical calculations validated the good ability of our CoMFA and CoMSIA model to predict the external dataset.

### CoMFA and CoMSIA contour map analysis

**Figure 4:**
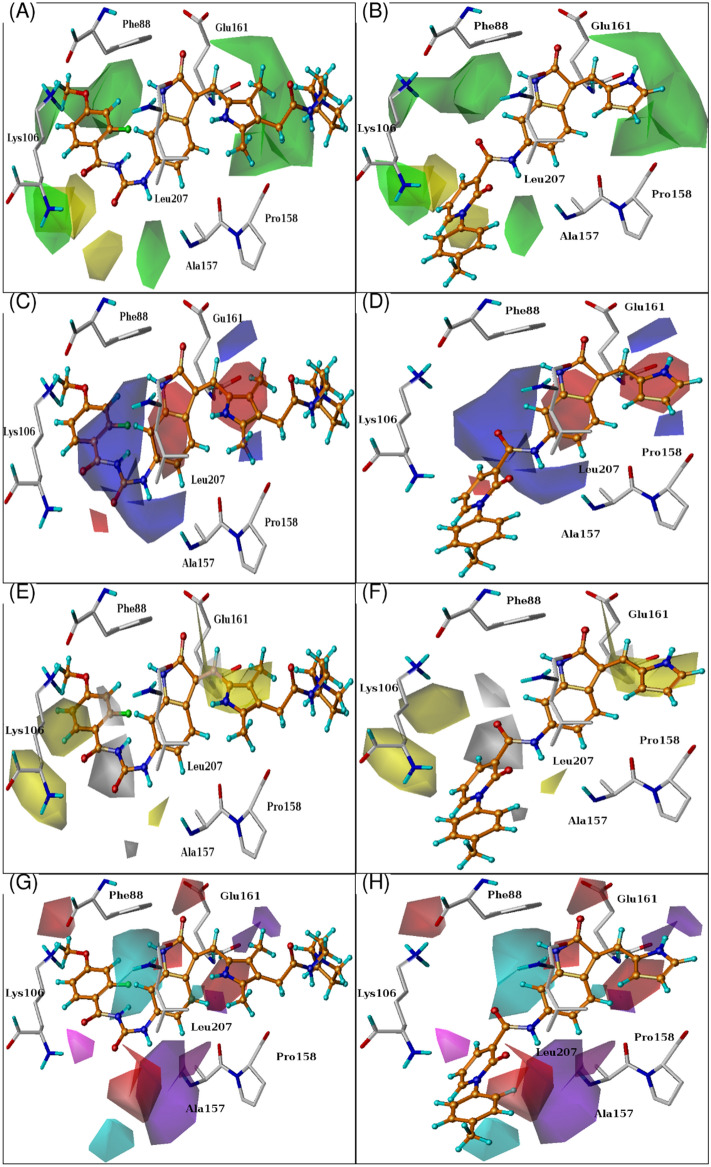
CoMFA and CoMSIA contour maps with combination of active and least active compounds. The active site residues, most active and inactive compound are presented for the comparison of their position with respect to contour maps position. The steric contour depicted in green and yellow contour indicates sterically favour and disfavour group (**A,B**). Electrostatic maps depicted in red and blue color shows favoured level for electron withdrawing and donating group respectively (**C,D**). Similarly, Hydrophobic contour depicted in Yellow and white maps indicate favoured and disfavoured regions for hydrophobicity (**E,F**). Hydrogen bond donor field presented in cyan and purple maps indicates the favoured and disfavoured areas for donating substituents. Hydrogen bond acceptor field presented in magenta and red contour indicates the favoured and disfavoured areas for hydrogen bond acceptor group (**G,H**).

To visualize the effects of CoMFA and CoMSIA fields on acyl and indoline derivatives in three dimensional spaces, the contour plots for the final model of CoMFA and CoMSIA are shown in Fig. 4. The contour analysis might be effective in recognizing the significant areas where changes in all five fields around the molecule describe the differences in IC_50_ values. The field type “standard deviation and coefficient” (stdev*-coeff) was used for building the contour maps. The compound acyl-54 (most active compound) was overlaid on contour plots for visualization.

In CoMFA model, the sterically favorable and unfavorable areas are highlighted by green and yellow colors respectively. While electrostatic map of CoMFA model shows blue and red color for electron donating and withdrawing group respectively. The CoMFA contour map is shown in Fig. 4(A–D).

It is shown that the sterically favorable contour is present near the linker which is adjacent to the core structure of biologically most active compound (acyl-54) indicating that the bulky substitution at that position is favorable (Fig. 4A). This is observed for two compounds, acyl-52 and acyl-53. Another green contour found near the carbonyl group of linker region also explains the sterically favorable substitution at this position. One of the green contours found near the methoxy group of flouro-benzoyl around Phe88 indicates that the methoxy group at this position is favorable for the activity of indoline derivatives, while in the case of indoline-14, 24a, 24c, 24g, 25a, 25h many fold decrease in inhibition is observed due to absence of methoxy group as compared to most active compound. A yellow contour is present near the CH2 of NH(CH2)2pyrrolidin-1-yl methyl group which indicates that the less bulky substitution is suitable for this position.

The CoMFA electrostatic maps displayed in Fig. 4(C,D), represented by red and blue color contours, indicate electron withdrawing and donating groups respectively. Two red contours found near the carbonyl oxygen of the ureido moiety and the side chain the of pyrrole ring indicate that electron withdrawing group around these areas is responsible for increasing the AK-B inhibitory activity. This is verified experimentally by the compounds indoline 24a–24i and 25h where decreased in activity is observed in the absence of these carbonyl oxygens. A blue contour is found near the NH which is adjacent to 2-floro-4-methoxybenzoyl group which indicates that electron donating group at this position is favorable for increasing inhibitory activity. Presence of another red contour indicating that 2-fluoro substituent on 4-methoxy benzoyl was required for optimum activity of aurora kinase B. Similar results can be observed in case of compound acyl-32a, 32b, 32c, 34 and 35 that do not contain fluorine atom at the aforementioned position which is might be responsible for their reduced activity in comparison to acyl-54.

The steric and electrostatic contour map analysis of CoMSIA model showed similar results to CoMFA. Therefore, the remaining three fields of CoMSIA model (hydrophobic, hydrogen bond donor and acceptor) are discussed in this section. The hydrophobic contour analysis of CoMSIA model is represented in Fig. 4(E,F), in which white contour is favorable for hydrophilic character while yellow areas were favorable for hydrophobic features. A white contour was found near the NH of pyrrole ring demonstrating that the hydrophilic substitution at this position was favorable for enhancing its inhibitory activity. At the same time, another large white region was observed near ureido moiety which is complementary to the pocket requirement. A yellow contour was observed near methyl of pyrrole ring suggesting the significance of non-hydrophobic character at pyrrole ring for AK-B inhibition activity as shown in Fig. 4(E,F).

The hydrogen bond donor and acceptor analysis represented in Fig. 4(G,H), the cyan and purple contour represent the maps where hydrogen bond donor groups favored and disfavored the activity respectively. Two medium sized cyan contours were observed near NH of pyrrole ring and indoline scaffold indicating that it fulfils the pocket requirement and responsible for inhibitory activity of indoline derivatives. A purple contour was found around the methoxy group of 2-floro-4-methoxybenzoyl indicating that the hydrogen bond donor substituent is not favorable in this zone. The structure of compound 32a (pIC_50_ = 7.8) and 32b (pIC_50_ = 8.2) presented the similar results, the only difference in their structure is the replacement of methoxy group by hydrogen in 32b, which shows better activity than 32a.

The magenta and red contour of Fig. 4(G,H) revealed the areas where hydrogen bond acceptor groups increase and decrease activity, respectively. A red contour near NH of indoline core structure suggested that hydrogen bond acceptor group is unfavorable at this position which is also proved experimentally. Another red contour appeared near NH of ureido moiety, suggesting that the hydrogen bond donor groups are favorable at this position. Two magenta contours were found near the carbonyl oxygen of ureido moiety indicating the importance of a hydrogen bond acceptor group at this position for better activity. Here, compounds acyl-32a-32j, 34, 38–53 with ureido moiety show better activity than those which do not contain ureido moiety such as indol-14-14b, 24a, 25h. The biological activities of these compounds are lower, consistent with the contour analysis, showing that our model is robust enough and can be used for future predictions.

### Identification of hits via structure-based pharmacophore

**Figure 5:**
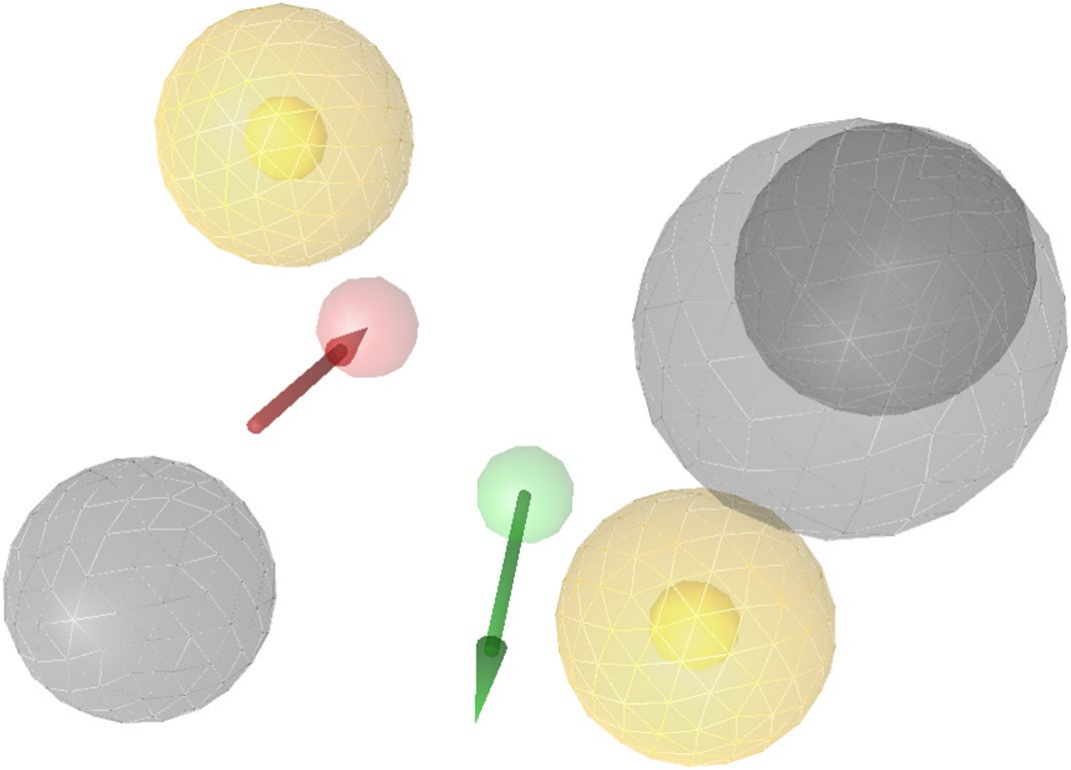
Selected structure-based pharmacophoric map is the combination of shared pharmacophores generated by 2BFY, 2VGP and 4AF3. The model comprised of seven features included two hydrophobic (yellow color), one donor (green vector), one acceptor (red vector) and three exclusion volume (grey color).

A pharmacophore can be defined as “a collection of electronic and steric features which is required for optimum molecular interaction with a specific biological target, to block its biological response^76^.” For successful structure-based pharmacophore virtual screening one should know the maximum information about target of interest. To meet this point, we developed a robust pharmacophore model by using eight crystal structures of AK-B to identify novel compounds that may inhibit AK-B activity. For this purpose, eight basic pharmacophore models were generated, then aligned to produce number of shared and merged pharmacophore models to identify the best model with true and high active hit rate. The selected model was produced by combination of 4AF3^51^, 2VGO^53^ and 2VGP^55^ features i.e. two hydrophobic (yellow color), one donor (green), one acceptor (red) and three exclusion volume (grey). The selected pharmacophore model with desired features is represented in Fig. 5. The generated pharmacophore model was then assessed for its capability to discriminate between true positive and true negative by actives, random and decoys dataset. The hit rate of the best model with these three datasets is 70%, 3% and 10% respectively. Compound libraries from ChemBridge^77^, MayBridge^78^, NCI^79^ and ZINC^80^ databases (> 30 million) were first filtered via drug-like and lead-like parameters to give 17.4 million molecules that were further decreased in number (16.9) by removing duplicates. The selected model was used to screen filtered databases and ~ 23,000 hits were obtained. The 70% of active lie in top 25% of the dataset which shows the reliability of pharmacophore model, these results were also supported by statistical techniques i.e., AUC and enrichment factor. ROC curve was calculated to measure the sensitivity and specificity between active and decoys in order to avoid false positive prediction on the basis of pharmacophore fitness score and the value of AUC was found to be 0.73. Further dataset was reduced by similarity signature technique via ROC software with 0.6 cut-off. After similarity search, > 2000 compounds were used for biological activity prediction by previous developed 3D-QSAR model. Finally, 17 compounds were considered as hits on the basis of pharmacophore fit score, docking interaction and 3D-QSAR predictions.

### Binding mode prediction for active compounds by docking

Molecular docking was performed using the Dock suite in MOE^48^ to predict the most plausible binding conformation of the compounds. Docking is a computational technique that aims to predict the binding position poses of small molecules in protein binding sites, with scoring functions used to evaluate which poses are best matches to the protein binding site. In this study, two aspects are analysed to measure the quality of docking method: first is docking program should be able to reproduce the experimentally determined crystal pose and second the scoring function should be able to discriminate between true binders and non-binders (active and inactive).

Primarily acylureidoindolin derivatives as potential Aurora-B inhibitors are docked in the active site, to investigate the binding site of AK-B.

**Figure 6:**
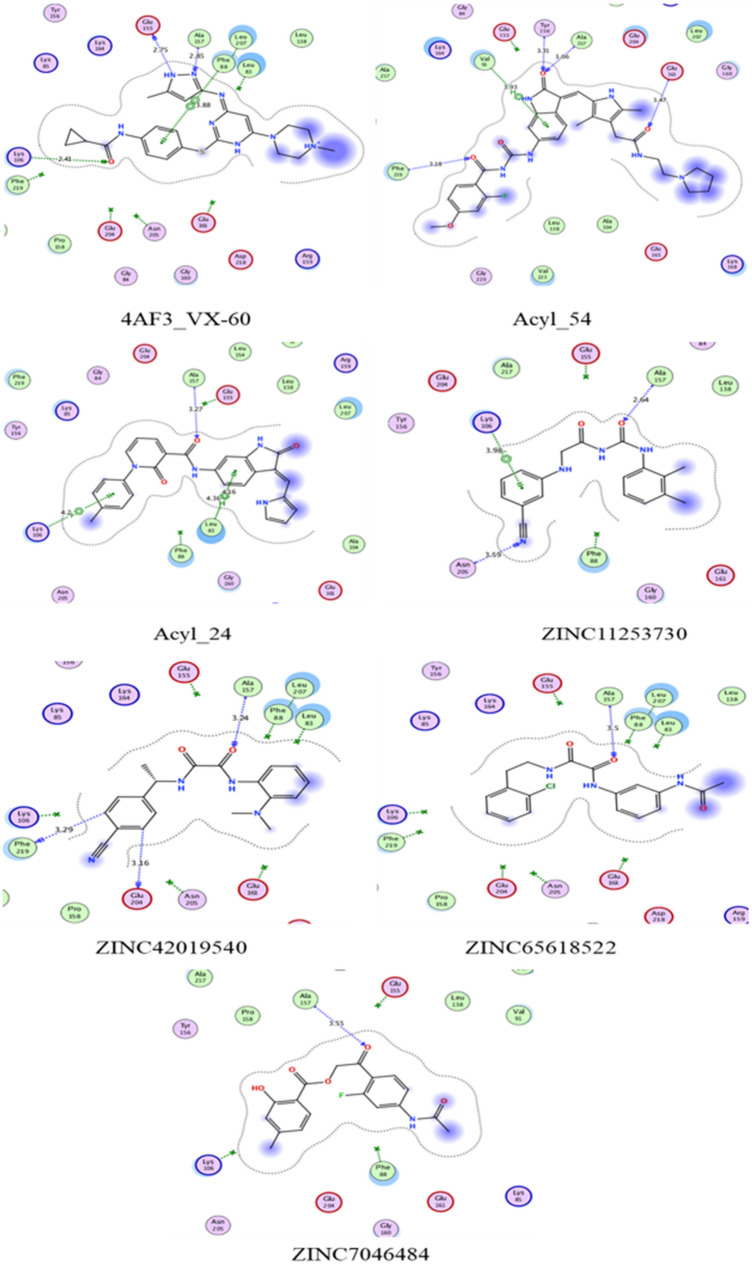
2D Ligand interaction diagrams for the top scored pose of selected compounds for docking into the binding site of the AK-B using MOE software; non-polar and polar residues are presented in green and pink circles; hydrogen bond interaction is indicated by green dotted arrows; Shape of the binding site indicated by the proximity contour (dotted lines) surrounding the ligand and available space to the more outward-facing parts of the ligand.

After prediction of hit compounds by the 3D-QSAR model, seventeen compounds were selected based on pharmacophore fit score, docking scores (London dG and GBVI/WSA dG) and prediction by 3D-QSAR models. As the most representative sample, the binding mode of AK-B (PDB ID: 4AF3^51^) was selected to continue with further docking analysis. This analysis included assessing the compounds position inside the binding cavity and their hydrogen bond formation pattern. It is evident that these compounds bind in the catalytic pocket of AK-B. The interactions specifically with Lys106, Glu155 and Ala157 in the hinge region and Leu83, Val91 and Leu207 in the conserved hydrophobic region are consider for the binding affinity. Only four compounds from the ZINC database showed hydrogen bond interactions with Lys106, Glu155 and Ala157 as shown in the Fig. 6. The proposed binding modes of the compounds ZINC11253730, ZINC42019540, ZINC65618522 and ZINC7046484 are shown in Fig. 6. Analysis of the binding mode of compound ZINC11253730 showed that it interacted via hydrogen bond between the carbonyl oxygen of the carbamoyl acetamide and the NH of Ala157 with the distance of 2.64 Å. Another hydrogen bond was observed between the side chain of Glu155 and carbonyl oxygen of ZINC11253730 at a distance of 2.36 Å. Asn206 also attached via hydrogen bond that radiated from its side chain oxygen and targeted the CN of compound with the distance of 3.5 Å. Besides hydrogen bonds, the molecule also interacted to the target protein via hydrophobic interaction through its benzonitrile ring to the phenyl ring of Lys106. Similarly, compounds with zinc identification code ZINC65618522 and ZINC7046484 interacted efficiently with Ala157 and Glu155 through hydrogen bond of ~ 3.0 and ~ 2.5 Å respectively. These compounds also bound through hydrophobic interaction via their phenyl ring with the five-membered ring of Pro158. Another compound, ZINC42019540 (which is similar to ZINC11253730) interacted through two hydrogen bonds with the backbone NH of Ala157 and carbonyl oxygen of compound mentioned in the Fig. 6, however, Leu83, Phe88, Pro158 and Leu207 residues formed CH-π and π-π interactions with all these compounds.

### Stability of complexes and binding mode analysis

**Figure 7:**
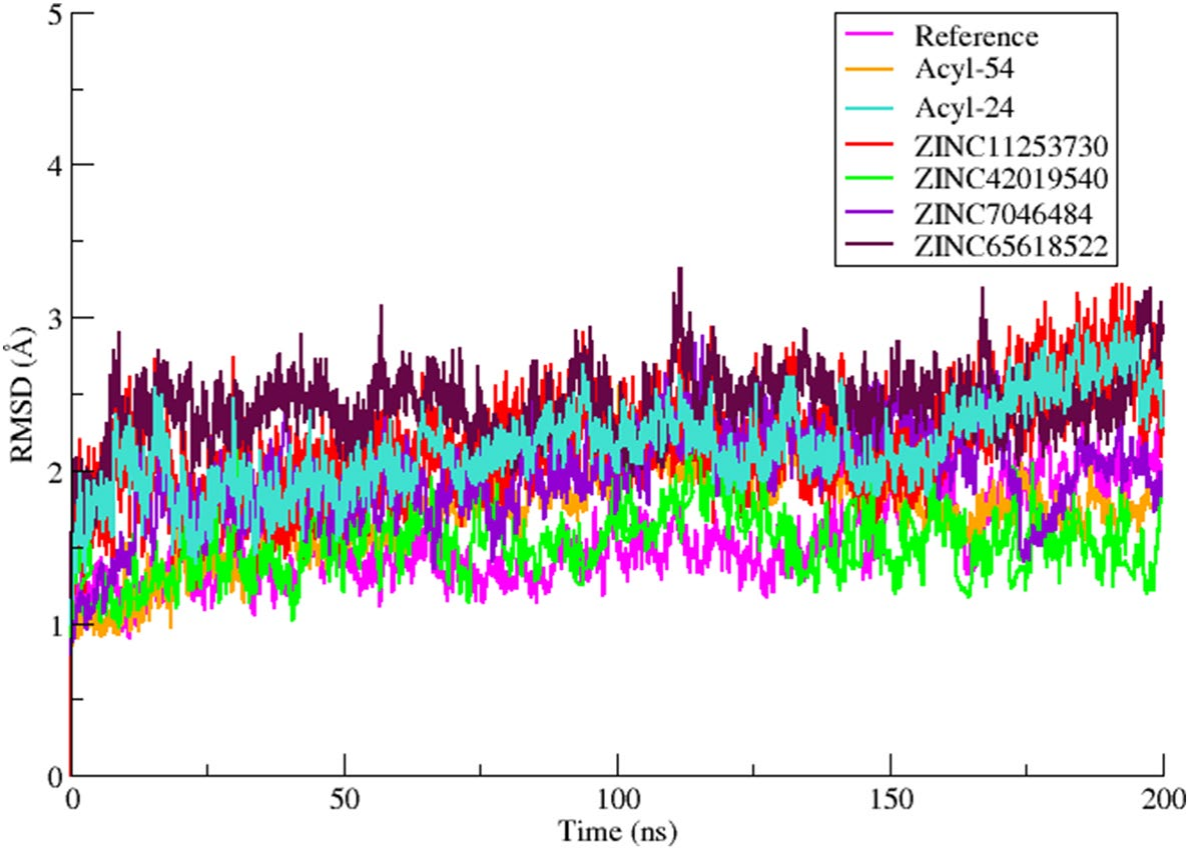
The CαRMSD of the MD runs of the six selected AK-B inhibitor complexes (Acyl-54(active), Acyl-24(inactive), ZINC11253730, ZINC42019540, ZINC65618522, ZINC07046484) along with the reference complex VX-680 during the 200 ns MD simulations.

**Figure 8:**
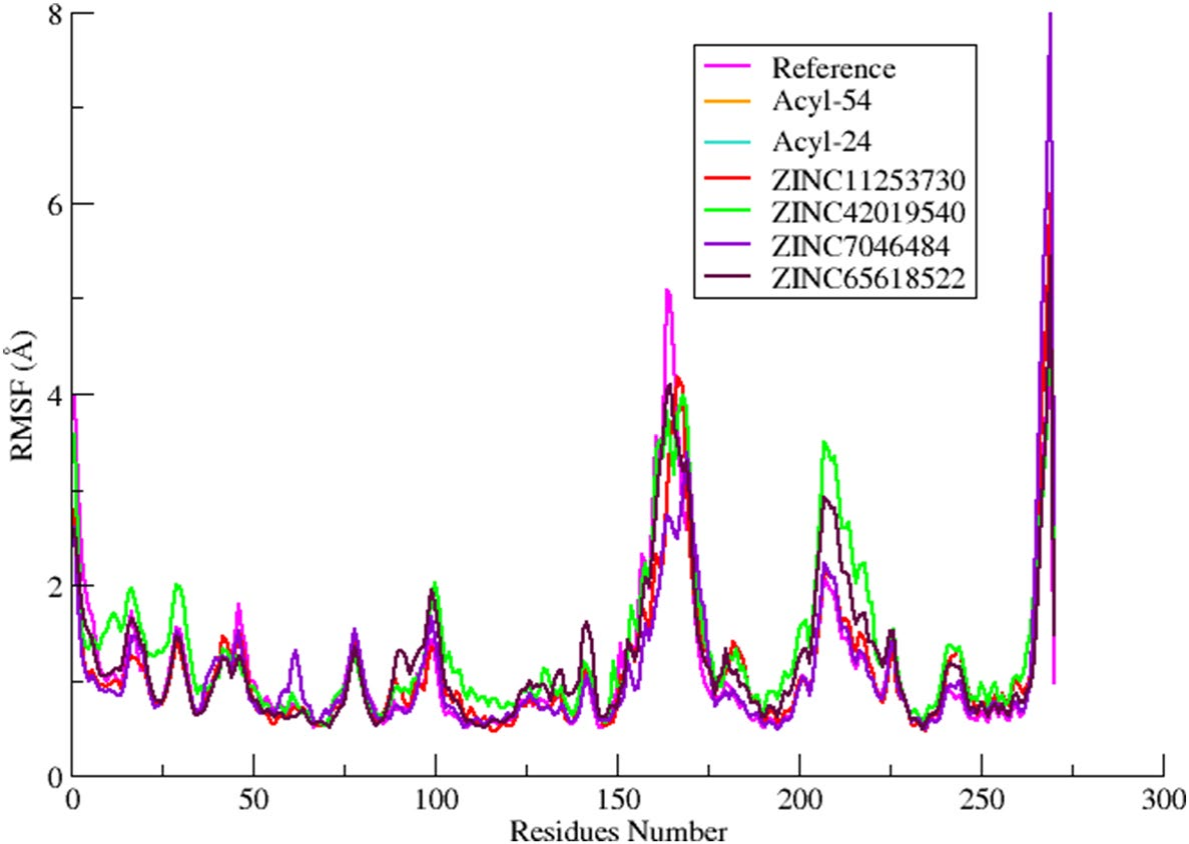
The CαRMSF of the MD runs of the six selected AK-B inhibitor complexes (Acyl-54(active), Acyl-24(inactive), ZINC11253730, ZINC42019540, ZINC65618522, ZINC07046484) along with the reference complex VX-680 during the 200 ns MD simulations.

Initially, replicates of 200 ns MD simulations were performed with both forcefields to explore the binding of the reported inhibitors to AK-B protein. In this regard a total of eight simulations were performed using crystal complex (4AF3) and the reported most active inhibitor (Acyl-54) of acyl ureido indoline derivative. The results indicate that ff14SB force field is more suitable for AK-B system than ff99SB due to excessive structural drift in the activation loop of AK-B. Detailed results for both forcefields are provided in the SI (Figure S2–S3). After analyzing the results with both force fields, the ff14SB force field was finally used to run the 200 ns MD simulations of remaining complexes with Acyl-24, ZINC11253730, ZINC42019540, ZINC65618522 and ZINC07046484. Simulation convergence and system stability were monitored by computing root mean square deviation (RMSD) of backbone atoms C, Cα, N and O with respect to the starting structure (Fig. 7). The RMSD plot showed stability of all systems at around 5 ns. The RMSD values of all systems initially increased during equilibration phase and indicate convergence after 10 ns. The active site residues show stability with less deviation. The RMSF plot was also analyzed from 0 to 200 ns for the regions which show higher flexibility (Fig. 8). The largest fluctuations were observed in the loop region. The binding of compounds did not affect the proteins overall conformational diversity. The interaction analysis in terms of hydrogen bonding and hydrophobic interactions and their occupancy were calculated during simulation (Figure S4–S5).

The reference compound VX-680 binds deeply within the active site of AK-B via a number of hydrophilic and hydrophobic interactions with the residues Lys106, Glu155 and Ala157 (hinge region) and Leu83, Val91 and Leu207 of hydrophobic region respectively. The amino group linking the pyrazole and pyrimidine ring show hydrogen bonding to the carbonyl oxygen atom of Ala157 throughout simulation with 99% occupancy. Another hydrogen bond which is not found during docking is observed between the Ala157 and N atom of pyrazole ring. The rest of the hydrophobic interactions observed in docking remain stable during the MD simulation. The benzene ring of VX-680 forms a pi–pi interaction with residue Phe88. Initially the cyclopropyl ring formed hydrophobic interaction with Leu207 but after 2 ns simulation it projected out towards the solvent. The piperazine ring is placed in a solvent-exposed region of the cavity and encircled by Arg81, Leu83, and Gly160. In case of the most active compound acyl-54, most of the hydrophobic interactions found by docking were conserved during MD simulation, while changes were observed for a number of hydrogen bonds. The NH of Ala157 formed hydrogen bond with double bonded oxygen of indoline-2-one moiety with 100% occupancy. Another new hydrogen bond was observed during MD simulation between the linker oxygen of acyl-54 and the sidechain of Arg82. The strong interaction of important residues with acyl-54 suggested its contribution to their inhibitory activity. Similarly, for compounds ZINC65618522 and ZINC7046484, hydrophobic interactions are conserved during MD simulation while hydrogen bonds with Glu155 and Ala157 remain unstable throughout the MD simulation. The phenyl ring of both compounds undergoes hydrophobic interaction with the side chain residues Phe88, Leu83, Lys106, Pro158 and Leu207. ZINC11253730 and ZINC42019540 showed similar interactions with the active site of AK-B. The carbonyl oxygen of benzamide is found in the vicinity of hinge region and forms hydrogen bond with the backbone and side chain of Ala157 and Glu155 respectively. The benzonitrile ring of ZINC42019540 shows CH- π and T-shaped π–π stacking interaction with Phe219. In the case of ZINC11253730, this CH–π interaction was found between benzonitrile ring and Lys106.

### Binding free energy calculations

**Figure 9:**
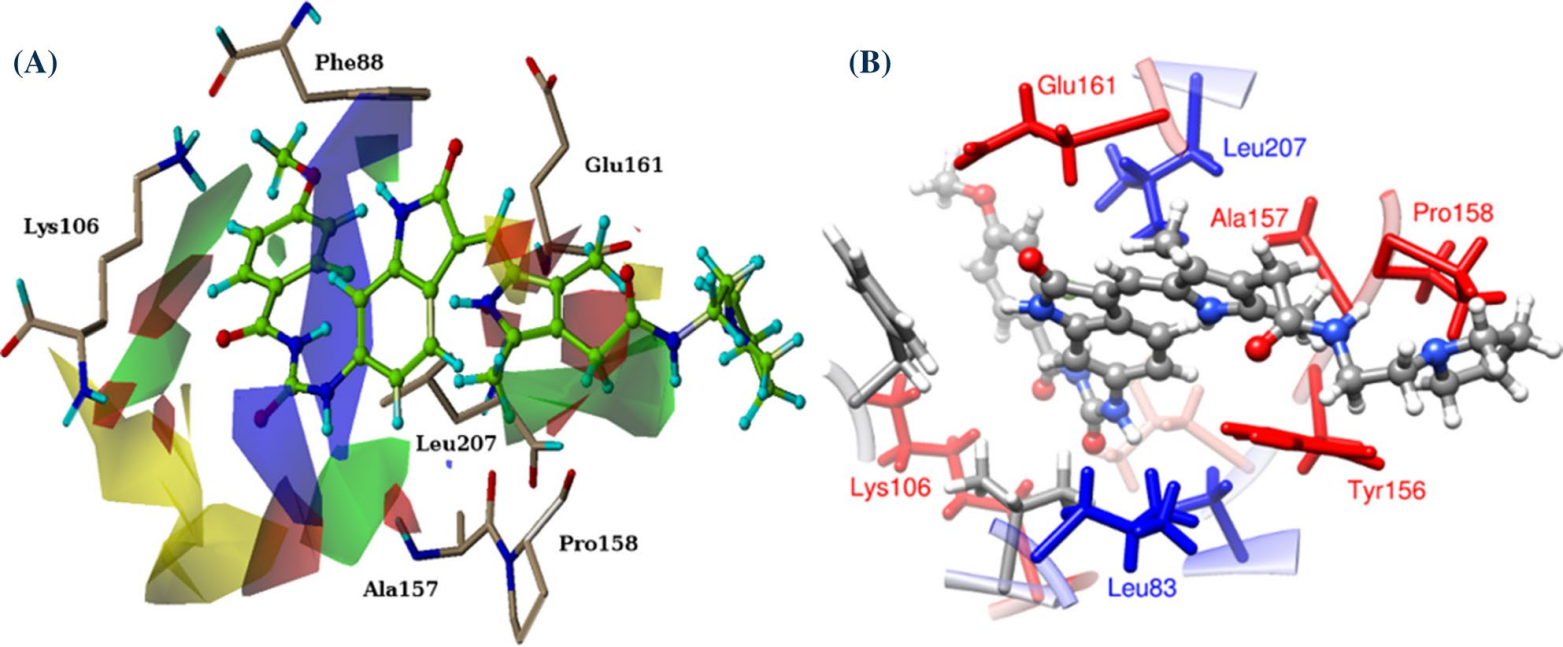
Comparison of 3D-QSAR (left) and WaterSwap(right) maps. The WaterSwap map shows the contributions to the free energy, with red values indicating preference of that residue for the electrostatic interaction, and blue indicating residues that favour binding of the ligand by van der Waals interactions.

**Figure 10:**
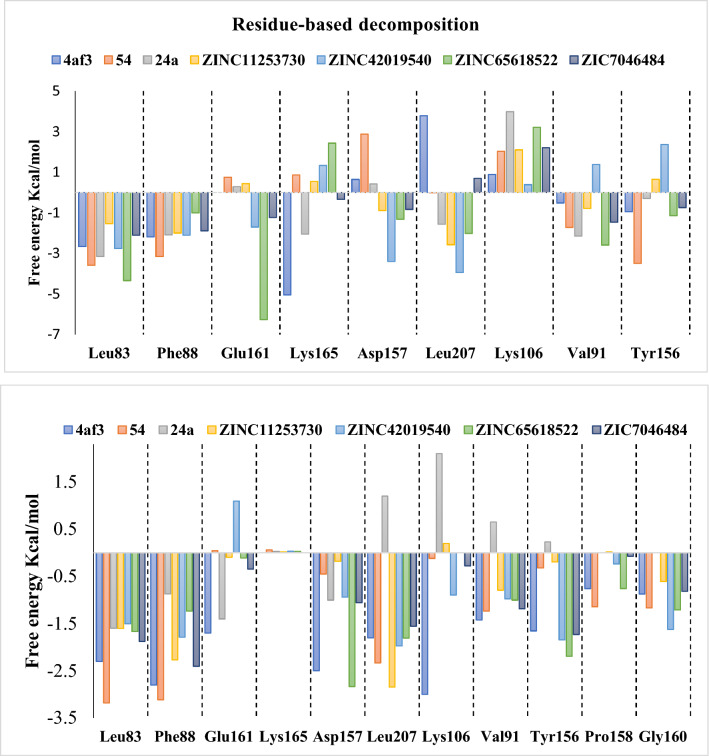
Upper panel: WaterSwap^45^ residue energy contributions. Positive values indicate stabilization of the water cluster, while negative values indicate stabilization of the protein–ligand complex. Lower panel indicate the MMGBSA decomposition results of total binding free energy per residue.

The interactions were also analyzed by calculation of absolute binding free energies using the WaterSwap^45,64,81–87^ and MM(GB/PB)SA^65^ methods. Generally, the MM(GB/PB)SA method is used to compute the binding free energy by selecting snapshots at regular intervals from the trajectory of entire MD simulation. This method does not consider the protein: solvent and ligand: solvent interaction details as it uses an implicit water model. Information concerning bridging interactions of solvent molecule between protein and ligand is of great importance. This problem can be avoided by using advanced and more convenient assay of WaterSwap, which uses explicit water model, so does not suffer with such limitations. The WaterSwap and MMGBSA calculations produce binding free energies in the range of − 20 to − 35.3 and − 36.2 to − 14 kcal/mol, respectively indicating that all inhibitors bind strongly to the AK-B protein (Table 4). For the selected AK-B inhibitor complexes, the range of IC_50_ is very narrow which is challenging for computational methods. If VX-680 is ignored as an outlier, then the docking score show good correlation coefficient with MMGBSA and WaterSwap with correlation value 0.58 and 0.68 respectively. A good correlation (R = 0.662) is observed between MMGBSA and WaterSwap, if compound ZINC42019540 is removed as an outlier. Overall, the binding free energies obtained from WaterSwap, MMGBSA and MMPBSA do not differ significantly. If VX-680 is ignored as an outlier then the MM(GB/PB)SA results predict the correct order of the compounds. The most potent compound showed results similar to the reference compound in case of MM(GB/PB)SA method. This might be because of hydrogen bonding to the hinge region which is necessary for the inhibition of kinases^73^. WaterSwap absolute binding free energy calculations for all seven compounds were performed on five representing structures after clustering of 200 ns MD simulation. The resulting binding free energy per compound obtained from FEP, Bennett and TI method (which were combined to produce an average value) can be compared with the experimentally derived binding free energy. The average absolute binding free energy of all systems is presented in Table 4. Further, the binding free energy is decomposed into individual residue contributions (Figs. 9–10). The decomposition analysis, highlight the major contributing residues for binding of AK-B inhibitors. For visualization of this residue decomposition, the CHEWD^88^ plugin for Chimera was used.

**Table 4:** Binding free energies of the selected inhibitor-bound complexes (VX-680, ZINC11253730, ZINC42019540, ZINC65618522, ZINC07046484, Acyl54 and Acyl24) from WaterSwap and MM/(GB/PB)SA calculations.

Inhibitor-bound complex	WaterSwap							
	IC_50_ nM	MOE Score kcal/mol	MM/PBSA kcal/mol	MM/GBSA kcal/mol	BAR kcal/mol	FEP kcal/mol	TI kcal/mol	Average kcal/mol
VX-680	− 10.3	− 9.6	− 32.4 ± 0.25	− 36.2 ± 0.23	− 35.1	− 34.9	− 35.8	− 35.3 ± 0.3
ZINC11253730	− 11.4	− 10.2	− 26.2 ± 0.31	− 29.1 ± 0.28	− 24.8	− 23.7	− 24.2	− 24.2 ± 0.4
ZINC42019540	− 11.0	− 10.4	− 16.3 ± 0.24	− 20.0 ± 0.31	− 30.6	− 29.9	− 30.9	− 30.5 ± 0.4
ZINC65618522	− 11.2	− 9.7	− 22.6 ± 0.28	− 25.5 ± 0.25	− 25.4	− 24.8	− 25.6	− 25.3 ± 0.3
ZINC07046484	− 10.3	− 9.8	− 15.4 ± 0.34	− 19.9 ± 0.24	− 25.1	− 25.5	− 24.6	− 25.1 ± 0.4
Acyl54	− 11.5	− 10.8	− 31.8 ± 0.32	− 36.0 ± 0.26	− 28.4	− 27.6	− 27.5	− 27.8 ± 0.4
Acyl24	− 4.5	− 6.5	− 8.19 ± 0.27	− 13.9 ± 0.35	− 20.7	− 19.8	− 20.0	− 20.1 ± 0.3

Here, the WaterSwap residue-based binding energy decompositions were investigated to highlight the residue which significantly contributed to the inhibitor binding throughout the course of MD simulations. The time-averaged values of the total (Δ*G*_residue_) components for all crucial residues that display good binding affinity towards the short listed compounds are shown in the Fig. 10. The Residues with positive values from the WaterSwap decomposition analysis favour binding of the water cluster, while negative values indicate that the residue contributes favourably to ligand binding. This shows that in case of reference compound 4af3, acyl-54 and acyl-24 charged residues (Lys106 and Glu161) stabilise the water cluster, while these compounds are stabilised by promising hydrophobic contacts with the neighbouring residues Leu83, Phe88, Val91, Pro158, Gly160 and Leu207 of the binding site, and all these come into direct contact with the large compounds, as some of these contacts are missing in case acyl-24 which is the least compound of the series and also smaller in size. This is realized that the binding contribution is increasing with ligand size. Similarly, the short-listed compounds ZINC11253730, ZINC42019540, ZINC65618522 and ZINC7046484, are stabilized by hydrophobic contact with the surrounding residues Leu83, Phe88, Tyr156, Leu207 and Phe219. Besides hydrophobic interactions, these compounds also show negative contribution with the charged residues Glu155 and Ala157 because of their involvement in hydrogen bond interaction. The interaction between the compound and Lys165 is unfavourable in all complexes except 4af3 and acyl-54, where it is slightly favourable due to its participation in van der Waals interactions. After computing residue-based binding free energy decomposition, the results of WaterSwap and 3D-QSAR maps were correlated. For this purpose, the components of Δ*G*_residue_ value were visualised by modifying them into a score which is used to colour each corresponding residue differently in molecular viewer. Snapshot from the resulting movie of acyl-54 (most active compound) are shown in Fig. 9. The trajectory displays that the residue based free energy components are fairly stable throughout the simulation. The residues are coloured by their total, van der Waals and electrostatic free energy components.

Additionally, Fig. 9 shows that the charged residues Lys106, Glu155 and Ala157 in the hinge region provides strong electrostatic stabilization (highlighted in red); this result is consistent with the 3D-QSAR maps in which two red contours found near the carbonyl oxygen of the ureido moiety of acyl-54 indicate that electron withdrawing group around these areas is responsible for increasing the AK-B inhibitory activity. Additionally, the compound attains promising van der Waals stabilisation by residues Leu83, Val91 and Leu207 (shown in blue). This decomposition reveals the experimental observation that the compound mostly binds via hydrophobic interactions. This observation is also well correlated with 3D-QSAR maps. The decomposition result also indicates that binding affinity of compound may be improved by adding a hydrogen bond donor group to get the same hydrogen bonding interactions that are observed to stabilise the swapped water cluster. To best of our knowledge, this study provides the first comparison of WaterSwap fields and 3D-QSAR maps.

To get further insight into inhibitor binding, MM/GBSA free energy decomposition analysis was used to decompose the total binding free energies into per residue components (Fig. 10). Table 4 lists the binding free energies for all inhibitor complexes. The residue decomposition approach suggested that major binding contribution comes from Glu155, Trp156 and Ala157 of site B and Leu83 and Leu207 of site C that play an important role in the binding. Free energy decomposition also shows favourable electrostatic contributions by Glu155 and Ala157 in both the WaterSwap and 3D-QSAR results.

## Conclusions

AK-B is a promising target in the field of oncology^7^. The current study employed structure-based pharmacophore modelling and an atom-based 3D-QSAR analysis of acylureidoindolin derivatives as AK-B inhibitors, followed by MD simulation and free energy calculations. The selected CoMFA and CoMSIA model exhibited good extrapolative power and strong correlation between theory and experiment with cross-validation values (q^2^) of 0.68, 0.641 and linear regression values (r^2^) of 0.971, 0.933 respectively. The contour maps of 3D-QSAR indicate that electrostatic and hydrophobic fields explain the activity of acylureidoindolin derivatives. Structural requirements including the carbonyl group of ureido moiety and the methoxy of the fluorobenzoyl in the vicinity of Phe88 are important for activity. Multiple crystal structures (Table 1) of AK-B inhibitor complexes were used to generate structure based pharmacophoric hypothesis to facilitate the identification of novel compound from commercial database. All compounds that satisfied the structural pharmacophoric features were analyzed with our 3D-QSAR model. To remove false positives, and refine the hits, 7 compounds were evaluated by MD simulation and binding free energy analysis. Decomposition of overall binding energy showed that major contributions came from Glu155, Trp156 and Ala157 of site B and Leu83 and Leu207 of site C. These results could help in understanding AK-B inhibition and in designing more specific and potent inhibitors against AK-B protein to treat various malignant disorders.

## Supplementary Information

Supplementary Information.

## Data Availability

Data created during this research is provided as supplementary information accompanying this paper.
