# Supplementary material for: Exploration of the structural requirements of Aurora Kinase B inhibitors by a combined QSAR, modelling and molecular simulation approach
Source: Sci Rep. 2021 Sep 21;11:18707. doi: 10.1038/s41598-021-97368-3 (PMC8455585; doi:10.1038/s41598-021-97368-3)
Supplement: Supplementary file 1 — Supplementary Information. [file 41598_2021_97368_MOESM1_ESM.docx]

Table S1: The training and test set of compounds used to build the 3D-QSAR, with their reported biological activity (IC_50_) in nanomolar^30,31^

| Compounds | R_1_ | R_2_ | R_3_ | R_4_ | R_5_ | R_6_ | R_7_ | IC_50_ |
| --- | --- | --- | --- | --- | --- | --- | --- | --- |
| Indoline11A | H | H | H | H |  | H | H | 25.0 |
| Indoline11B | H | H | H | H |  | H | H | 29.6 |
| Indoline12 | CH_3_ | COOH | CH_3_ | H |  | H | H | 5.9 |
| Indoline13 | CH_3_ | NH(CH_2_)_2_pyrrolidin-1-yl | CH_3_ | H |  | H | H | 93.0 |
| Indoline14 | H | H | H | H |  | H | H | 252.6 |
| Indoline14B | H | H | H | H |  | H | H | 347.0 |
| Indoline24A |  | H | H | H |  | H | H | 791.1 |
| Indoline24B |  | H | H | H |  | H | H | 209.3 |
| Indoline24C | H | H | H | H |  | H | H | 87.4 |
| Indoline24D | H | H | H | H |  | H | H | 209.4 |
| Indoline24G | H | H | H | H |  | H | H | 250.6 |
| Indoline24H | H | H | H | H |  | H | H | 102.2 |
| Indoline24I | H | H | H | H |  | H | H | 32.5 |
| Indoline25A | CH_3_ | COOH | CH_3_ | H |  | H | H | 10.7 |
| Indoline25H | CH_3_ | COOH | CH_3_ | H |  | H | H | 14.3 |
| Indoline31A | H | H | H | H |  | H | H | 127.6 |
| Indoline31B | H | H | H | H |  | H | H | 10.2 |
| Indoline31C | H | H | H | H |  | H | H | 33.1 |
| Indoline31D | H | H | H | H |  | H | H | 63.2 |
| Indoline31E | H | H | H | H |  | H | H | 53.8 |
| Indoline31F | H | H | H | H |  | H | H | 348.0 |
| Indoline31G | H | H | H | H |  | H | H | 161.4 |
| Indoline31H | H | H | H | H |  | H | H | 25.5 |
| Indoline31I | H | H | H | H |  | H | H | 29.2 |
| Indoline32 | CH_3_ |  | CH_3_ | H |  | H | H | 4.5 |
| Indoline33 | CH_3_ |  | CH_3_ | H |  | H | H | 70.5 |
| Indoline34 | CH_3_ |  | CH_3_ | H |  | H | H | 91.6 |
| Indoline35 | CH_3_ |  | CH_3_ | H |  | H | H | 2.9 |
| Indoline36 | CH_3_ |  | CH_3_ | H |  | H | H | 73.9 |
| Acyl-32A | H | H | H | H |  | H | H | 14.9 |
| Acyl-32B | H | H | H | H |  | H | H | 5.3 |
| Acyl-32C | H | H | H | H |  | H | H | 6.5 |
| Acyl-32D | H | H | H | H |  | H | H | 7.7 |
| Acyl-32E | H | H | H | H |  | H | H | 6.5 |
| Acyl-32F | H | H | H | H |  | H | H | 40.9 |
| Acyl-32G | H | H | H | H |  | H | H | 15.0 |
| Acyl-32H | H | H | H | H |  | H | H | 18.4 |
| Acyl-32I | H | H | H | H |  | H | H | 29.8 |
| Acyl-32J | H | H | H | H |  | H | H | 158.7 |
| Acyl-34 | CH_3_ | COOH | CH_3_ | H |  | H | H | 1.9 |
| Acyl-35 | CH_3_ | H | CH_3_ | H |  | H | H | 55.5 |
| Acyl-38 | CH_3_ | CH_2_CO_2_H | CH_3_ | H |  | H | H | 1.8 |
| Acyl-39 | CH_3_ | (CH_2_)_2_CO_2_H | CH_3_ | H |  | H | H | 1.2 |
| Acyl-40 | CH_3_ |  | CH_3_ | F |  | H | H | 1.5 |
| Acyl-41 | CH_3_ |  | CH_3_ | F |  | H | H | 3.0 |
| Acyl-42 | CH_3_ |  | CH_3_ | H |  | H | H | 2.3 |
| Acyl-43 | CH_3_ |  | CH_3_ | F |  | H | H | 3.0 |
| Acyl-44 | CH_3_ |  | CH_3_ | H |  | H | H | 1.3 |
| Acyl-45 | CH_3_ |  | CH_3_ | F |  | H | H | 1.5 |
| Acyl-47 | CH_3_ |  | CH_3_ | H |  | H | H | 217.9 |
| Acyl-48 | CH_3_ |  | CH_3_ | H |  | H | H | 30.6 |
| Acyl-49 | CH_3_ |  | CH_3_ | H |  | H | H | 18.4 |
| Acyl-50 | CH_3_ |  | CH_3_ | H |  | H | H | 16.8 |
| Acyl-51 | CH_3_ |  | CH_3_ | H |  | H | H | 16.4 |
| Acyl-52 | CH_3_ |  | CH_3_ | H |  | H | H | 10.5 |
| Acyl-53 | CH_3_ |  | CH_3_ | H |  | H | H | 7.6 |
| Acyl-54 | CH_3_ |  | CH_3_ | H |  | H | H | 0.4 |


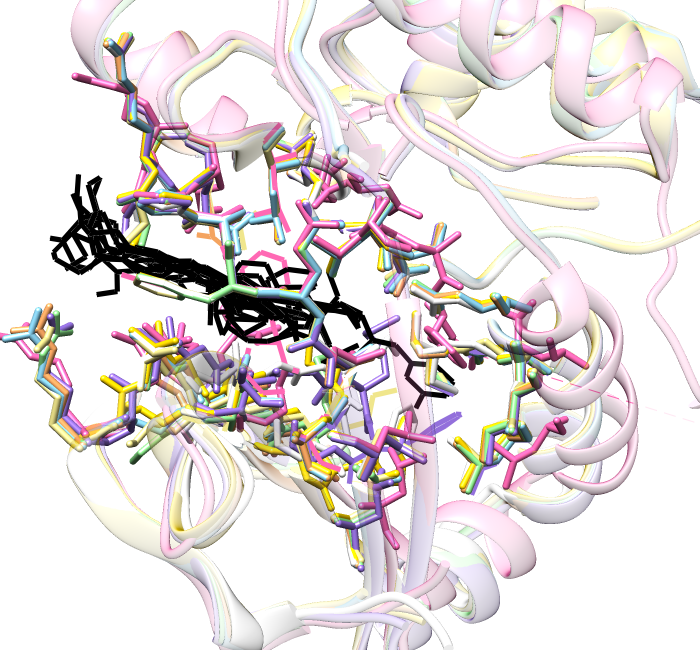


Figure S1: Alignment of all crystal structures of AK-B protein.




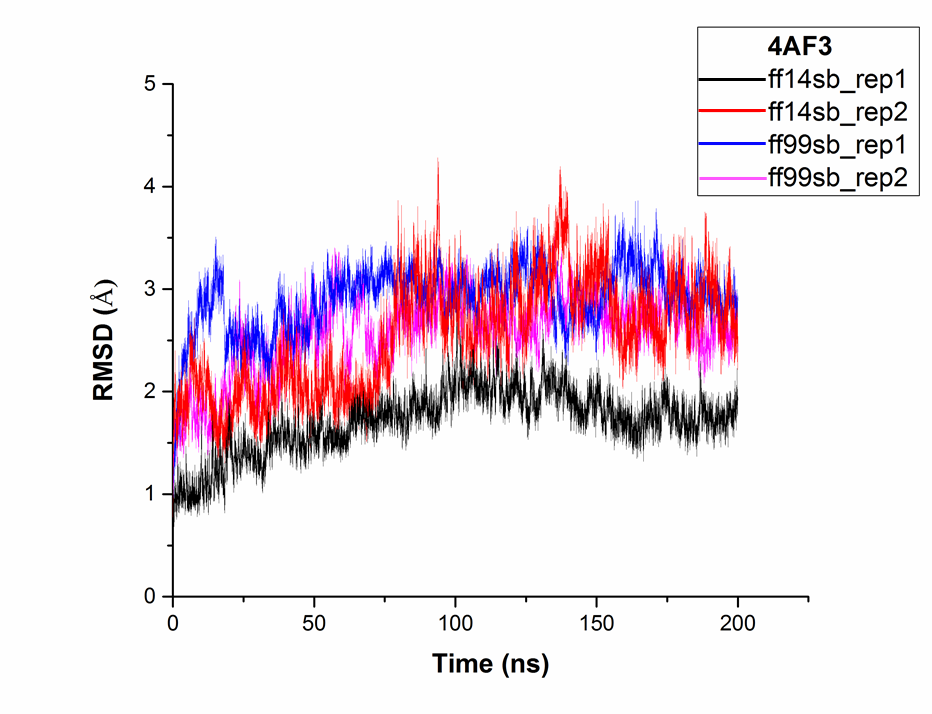


Figure S2: RMSD and RMSF plots of FF14SB and FF99SB force field of crystalized complex inhibitor of AK-B







Figure S3: RMSD and RMSF plots of FF14SB and FF99SB force field of Acyl-54 (most active compound of acyl ureido indoline derivatives) of AK-B

Figure S4: The frequency plot of hydrophobic contacts of AK-B binding site residues with crystallized inhibitor, Acyl-54, Acyl-24, ZINC11253730, ZINC42019540, ZINC65618522 and ZINC07046484.

Figure S5: Hydrogen bond occupancy plot of crystallized inhibitor, Acyl-54, Acyl-24, ZINC11253730, ZINC42019540, ZINC65618522 and ZINC07046484 with the crucial residues of AK-B binding site.
